# Avacopan for ANCA-associated vasculitis with hypoxic pulmonary haemorrhage

**DOI:** 10.1093/ndt/gfae020

**Published:** 2024-01-24

**Authors:** Aglaia Chalkia, Oliver Flossmann, Rachel Jones, Jagdish Ramachandran Nair, Thomas Simpson, Rona Smith, Lisa Willcocks, David Jayne

**Affiliations:** Department of Medicine, University of Cambridge, Cambridge, UK; Department of Nephrology, Royal Berkshire Hospital, Reading, UK; Department of Medicine, University of Cambridge, Cambridge, UK; Vasculitis & Lupus Clinic, Addenbrooke's Hospital Cambridge, Cambridge, UK; Department of Rheumatology, Liverpool University Hospital, Liverpool, UK; Department of Respiratory Medicine, Lewisham Hospital, London, UK; Department of Medicine, University of Cambridge, Cambridge, UK; Vasculitis & Lupus Clinic, Addenbrooke's Hospital Cambridge, Cambridge, UK; Vasculitis & Lupus Clinic, Addenbrooke's Hospital Cambridge, Cambridge, UK; Department of Medicine, University of Cambridge, Cambridge, UK; Vasculitis & Lupus Clinic, Addenbrooke's Hospital Cambridge, Cambridge, UK

**Keywords:** ANCA, avacopan, hypoxia, pulmonary haemorrhage, vasculitis

## Abstract

**Background:**

Pulmonary haemorrhage with hypoxia caused by anti-neutrophil cytoplasmic antibody (ANCA)-associated vasculitis (AAV) has a high early mortality. Avacopan, an oral C5a receptor antagonist, is an approved treatment for AAV, but patients with pulmonary haemorrhage requiring invasive pulmonary ventilation support were excluded from the Avacopan for the Treatment of ANCA-Associated Vasculitis (ADVOCATE) Trial.

**Methods:**

A retrospective, observational, multicentre case series of AAV patients with hypoxic pulmonary haemorrhage, requiring oxygen support or mechanical ventilation, who received avacopan.

**Results:**

Eight patients (62.5% female), median age 64 years (range 17–80), seven with kidney involvement, median estimated glomerular filtration rate (eGFR) 11 (range 5–99) mL/min/1.73 m^2^, were followed for a median of 6 months from presentation. Seven were newly diagnosed (87.5%), five were myeloperoxidase-ANCA and three proteinase 3-ANCA positive. All had hypoxia, four requiring mechanical ventilation (three invasive and one non-invasive). Intensive care unit (ICU) stay for the four patients lasted a median of 9 days (range 6–60). Four received rituximab and cyclophosphamide combination, three rituximab and one cyclophosphamide. Four underwent plasma exchange and one received 2 months of daily extracorporeal membrane oxygenation therapy. Following the initiation of avacopan after a median of 10 days (range 2–40), pulmonary haemorrhage resolved in all patients, even the two who had 1 month of refractory pulmonary haemorrhage prior to avacopan. Additionally, after 1 month, the median prednisolone dose was 5 mg/day (range 0–50), with three patients successfully discontinuing steroid use. Two patients suffered serious infections, two discontinued avacopan, one permanently due to a rash and one temporarily after 3 months due to neutropenia. All patients survived and no re-hospitalization occurred.

**Conclusion:**

We report the use of avacopan as a component of the treatment for pulmonary haemorrhage with hypoxia in AAV. Despite the life-threatening presentations all patients recovered, but attribution of the positive outcomes to avacopan is limited by the concomitant therapies and retrospective observational design.

## Graphical Abstract

**Graphical Abstract:**
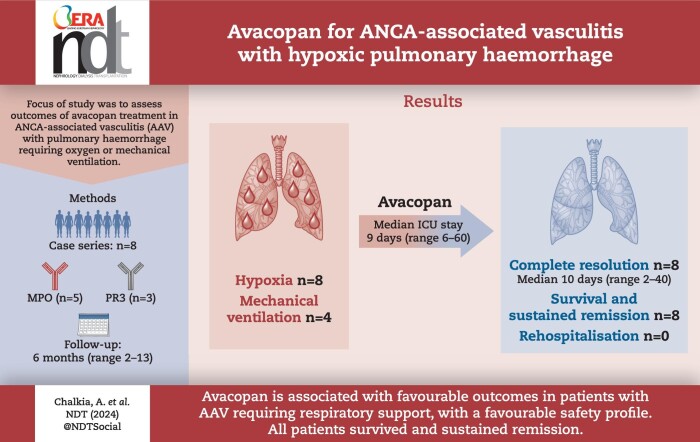

KEY LEARNING POINTS **What was known:**

- Pulmonary haemorrhage is a life-threatening manifestation of ANCA-associated vasculitis marked by a high risk of early mortality.
- Avacopan, an oral C5a receptor antagonist, has emerged as part of the induction treatment in combination with rituximab or cyclophosphamide.
- However, the ADVOCATE trial excluded patients with pulmonary haemorrhage requiring invasive pulmonary ventilation support.

**This study adds:**

- This case series describes the outcomes and safety of patients experiencing hypoxic pulmonary haemorrhage, including patients requiring mechanical ventilation, treated with avacopan.
- The outcomes observed appear promising, in terms of the rapid improvement in lung symptoms, faster tapering of steroid doses, and notably, the survival of all patients.

**Potential impact:**

- Our findings offer valuable insights and practical knowledge that can serve as an important initial reference for other physicians when managing similar cases.
- Further real-life data are required to inform clinical practice.

## INTRODUCTION

Anti-neutrophil cytoplasmic antibody (ANCA)-associated vasculitides (AAV) are characterized by small-vessel necrotizing inflammation and present with multisystem organ involvement, including organ/life-threatening manifestations such as pulmonary haemorrhage. AAV are divided into three clinical phenotypes of granulomatosis with polyangiitis (GPA), microscopic polyangiitis (MPA) and eosinophilic granulomatosis with polyangiitis (EGPA).

Each phenotype manifests a diverse range of pulmonary manifestations, with pulmonary haemorrhage being prominent in MPA (25%–60%) and GPA (22%–30%) [1]. In pulmonary haemorrhage the pathologic hallmark is a predominant neutrophilic inflammation, resulting in fibrinoid necrosis, primarily affected the pulmonary capillaries and disruption of the integrity of the alveolar–capillary membrane [2, 3]. Concomitant kidney involvement is frequent. While there have been advances in the management of AAV [4], patients still experience high mortality rates when compared with the general population [5]. In most cases, infections remain the leading cause of mortality [5, 6]. The Plasma Exchange and Glucocorticoids in Severe ANCA-Associated Vasculitis (PEXIVAS) Trial was the first randomized control trial that defined the clinical features of pulmonary haemorrhage in AAV and included patients with all severities of pulmonary haemorrhage. The trial subdivided pulmonary haemorrhage into ‘non-severe’ with no hypoxia, and ‘severe’ with hypoxia. Mortality was 23% at 3 months and 26% at 12 months in the ‘severe’ pulmonary haemorrhage group [7, 8].

Minimizing the adverse effects of treatment whilst achieving rapid disease control has been an important aim and has led to the development of avacopan, an oral C5a receptor antagonist, as an alternative to glucocorticoids [4]. The Phase 3 Avacopan for the Treatment of ANCA-Associated Vasculitis (ADVOCATE) trial compared avacopan in addition to rituximab or cyclophosphamide versus a glucocorticoid regimen, predominantly in a cohort with kidney involvement (80.7%) and less often with lung involvement (42.8%), excluding patients with life/organ threatening manifestations, such as pulmonary haemorrhage requiring invasive pulmonary ventilation support or estimated glomerular filtration rate (eGFR) <15 mL/min/1.73 m^2^ [9]. After avacopan gained regulatory approval, real-world clinical experience started to grow; however, there are a lack of reported data regarding its effectiveness and safety in patients with hypoxic pulmonary haemorrhage. In this context, we describe eight cases of AAV patients presenting with hypoxic pulmonary haemorrhage who were treated with avacopan as part of the induction treatment.

## MATERIALS AND METHODS

### Patients

We present a retrospective, observational, multicentre study that included patients with AAV who experienced pulmonary haemorrhage with hypoxia and received avacopan. These cases represent all the cases with pulmonary haemorrhage presented with hypoxia and received avacopan at a dose of 30 mg twice a day from four medical centres in the UK, between October 2022 and October 2023. The definition of pulmonary haemorrhage followed that of the PEXIVAS trial [7] namely: a compatible chest X-ray or computed tomography (CT) scan (diffuse pulmonary infiltrates) AND the absence of an alternative explanation for all pulmonary infiltrates (e.g. volume overload or pulmonary infection) AND one of the following: (i) evidence of alveolar haemorrhage on bronchoscopic examination or increasingly bloody returns with bronchoalveolar lavage; (ii) observed haemoptysis; (iii) unexplained anaemia (<10 g/dL) or documented drop in haemoglobin (>1 g/dL) from less than 10 g/dL; (iv) increased diffusing capacity of carbon monoxide. Hypoxia was defined as: an oxygen saturation of 85% or less while the patient was breathing ambient air or the use of mechanical ventilation.

### Data collection

All clinicians were required to complete a case report form for each patient, which included demographics data, clinical data, characteristics regarding the course of pulmonary haemorrhage, treatment characteristics, laboratory results and outcome.

In accordance with the UK National Health Service Research Ethics Committee guidelines, ethics approval was not required as this work comprised anonymous retrospective data and all treatment decisions were made prior to our evaluation.

### Statistical analysis

Descriptive statistics included medians with range (minimum value–maximum value) for continuous variables and frequencies (percentages) for categorical variables using Statistical Package for the Social Sciences (SPSS) software program, version 25.0 for Windows.

## RESULTS

### Clinical and treatment characteristics

The clinical and outcome characteristics are summarized in Tables 1 and 2. Eight patients (62.5% female) with a median age of 64 years (range 17–80) were included. Seven had a new diagnosis, while one had relapsed 1 year after their original diagnosis. Five were myeloperoxidase (MPO)-ANCA and three proteinase 3 (PR3)-ANCA positive. Seven had kidney involvement (Patient 7 had isolated pulmonary haemorrhage), characterized by a median eGFR 11 (range 5–99) mL/min/1.73 m^2^, while three were dialysis dependent at presentation.

**Table 1:** Clinical and treatment characteristics of avacopan treated patients with hypoxic pulmonary haemorrhage in ANCA-associated vasculitis.

Patient no., age/gender	Disease presentation, organ involvement	ANCA, MPO/PR3, levels (ref range)	Kidney involvement, eGFR, (mL/min/1.73 m^2^), ACR (mg/mmol)	Immunosuppressive treatment	PLEX Y/N, no. sessions	Avacopan, time of initiation	Steroids: οn avacopan initiation; 1 month after avacopan; discontinuation	Side-effects, after avacopan initiation
1, 65/F	New disease	MPO, 61 IU/mL (0.0–3.4)	• 20	Induction:	Y, 7	• 10th day	• 60 mg	N
	• Lung		• 340	• IV CYC (2 doses)			• Discontinuation	
	• Kidney			• IV RTX 1000 mg (2 doses)			• 30 days	
	• ENT			• IV GCs 500 mg ×3				
	• Constitutionalsymptoms			Maintenance:• IV RTX 1000 mg				
2, 17/M	New disease• Lung• PNS• Heart• Kidney	PR3, >177 IU/mL (0.0–1.9)	• CVVHDF• N/A	Induction:• IV CYC (4 doses)• IV RTX 500 mg ×5 weekly• IV IVIG• IV GCs[(500 mg ×3) ×3]Maintenance:• IV RTX 1000 mg	Y, 17	• 2nd month• Via nasogastric tube (ICU)	• 50 mg• 25 mg• 12 months	• Serious infections 1st month(bacterial and fungal)• Neutropenia (3rd month)Temporary discontinuation
3, 80/F	New disease• Lung• Kidney• ENT• Constitutionalsymptoms	MPO, 49 IU/mL (0.0–3.4)	• 9 (HD)• 451	Induction:• IV RTX 1000 mg (2 doses)• IV GCs 500 mg ×3Maintenance:• IV RTX 1000 mg	Y, 5	• 7th day	• 50 mg• 5 mg• 37 days	N
4, 78/M	New disease• Lung• Kidney• ENT	MPO, >134 IU/mL (0.0–3.4)	• 11• 45	Induction:• IV RTX 1000 mg (2 doses)• IV GCs 500 mg ×3Maintenance:Not yet received	N	• 5th day	• 40 mg• 5 mg• 60 days	Non-serious infection COVID-19 (20th day) treated with nirmatrelvir/ritonavir as outpatientReduction dose of avacopan
5, 63/F	New disease• Lung• Kidney• PNS	MPO, 27 IU/mL (0.0–3.4)	• 99• 5.6	Induction:• IV RTX 1000 mg (2 doses)• IV GCs 250 mg ×3Maintenance:• IV RTX 1000 mg	N	• 4th day• Discontinuation after 3 days	• 20 mg• Discontinuation• 35 days	• Rash (2nd day) (photosensitive), permanent discontinuation• Non-serious infection COVID-19 (4th month)
6, 65/F	New disease• Lung• Kidney• ENT• Skin• Constitutional symptoms	PR3, 105 IU/mL (0.0–1.9)	• 5 (HD)• N/A	Induction:• IV CYC (2 doses)• IV RTX 1000 mg (2 doses)• IV GCs 500 mg ×2Maintenance:Not yet received	N	• 1st day• Via nasogastric tube (ICU)	• IV 500 mg• Discontinuation• 32 days	Serious infections (1st and 2nd month) (bacterial)
7, 46/M	Relapsing disease• Lung• ENT	PR3, 14 IU/mL (0.0–4)	• 104Without kidney involvement	Induction:• IV CYC (2 doses)• IV RTX 1000 mg (2 doses)• IV GCs 1000 mg ×2Maintenance:• IV RTX 1000 mg	Y, 7	• 2nd month	• 20 mg• 7.5 mg• Remains on 7.5 mg	Non serious infection (6–7th month)
8, 60/F	New disease	MPO, 134 IU/mL (0.0–5)	• 74	Induction:	N	• 4th day	• 100 mg	N
	• Lung		• PCR = 25	• IV CYC (6 doses)			• 50 mg	
	• Kidney			• IV GCs 1000 mg ×3			• Remains on 7.5 mg	
				Maintenance:				
				Not yet received				

F, female; M, male; ENT, ear, nose, throat; PNS; peripheral nervous system; ref, reference; ACR, albumin–creatinine ratio (mg/mmol); PCR, protein–creatinine ratio (mg/mmol); CVVHDF, continuous venovenous hemodiafiltration; HD, haemodialysis; N/A, not available; IV, intravenous; CYC, cyclophosphamide; RTX, rituximab; GCs; glucocorticoids; Y, yes; N, no; ICU, intensive care unit.

**Table 2:** Overall outcome and efficacy of avacopan treated patients with hypoxic pulmonary haemorrhage in ANCA-associated vasculitis.

Patient	ICU Y/N, duration	Mechanical ventilation (invasive/non-invasive), duration	Oxygen requirement, duration, flow (max)	Haemoglobulin, lowest level (g/L)	Total duration of 1st hospitalization, re-hospitalization	Lung symptoms resolution^a^, duration	Total follow-up (months)	Survival	Last FU: eGFR (mL/min/1.73 m^2^), ACR (mg/mmol)
1	Y, 6 days	Non-invasive mechanical ventilation (6 days)	• 10 days• 15 L/min	79	• 16 days• N	Resolved, 13 days	9	Y	• 30• 54
2	Y, 60 days	Invasive mechanical ventilation (43 days), ECMO (60 days)	• 70 days• Mechanical ventilation	82	• 85 days• N	Resolved, 70 days	13	Y	• 140• 0.2
3	Y, 7 days	Invasive mechanical ventilation (4 days)	• 16 days• Mechanical ventilation	62	• 21 days• N	Resolved, 16 days	6	Y	• 5 (HD)• 98
4	N	N	• 16 days • 4 L/min	87	• 17 days• N	Resolved16 days	2	Y	• 24• 45
5	N	N	• 5 days• 3 L/min	76	• 9 days• N	Resolved, 7 days	6	Y	• 82• neg
6	Y, 11 days	Invasive mechanical ventilation (7 days)	• 11 days• Mechanical ventilation	53	• 85 days• N	Resolved, 32 days	3	Y	• 10 (HD)• N/A
7	N	N	• 2 days• 15 L/min	74	• 78 days• N	Resolved, 90 days	10	Y	• 107Without kidneyinvolvement
8	N	N	• 9 days• 15 L/min	92	• 10 days• N	Resolved, 9 days	5	Y	• 53• PCR = 20

a Off oxygen treatment, without haemoptysis and stable haemoglobulin.

Y, yes; N, no; max, maximum; N/A, not available; ACR, albumin–creatinine ratio (mg/mmol); neg, negative; PCR, protein–creatinine ratio (mg/mmol); FU, follow-up, HD, haemodialysis.

In six patients, avacopan treatment was initiated within the first 10 days after diagnosis of AAV. In two (Patients 2 and 7), avacopan was started later, between the first and second month, due to its unavailability during the early treatment period. Four received induction treatment with cyclophosphamide and rituximab, three rituximab and one cyclophosphamide. All patients received intravenous glucocorticoid pulses, with a cumulative dose of 750–4500 mg. Four underwent plasma exchange (PLEX), with a median of 7 sessions (range 5–17) and one required 2 months of continuous extracorporeal membrane oxygenation (ECMO) therapy for respiratory support.

### Pulmonary response

All but one patient presented with haemoptysis. Four required admission to the intensive care unit (ICU), with three requiring immediate ICU admission due to their critical presentation, and one patient was transferred to ICU 3 days after hospitalization. All four patients admitted to the ICU received mechanical ventilation, with three undergoing invasive ventilation and one non-invasive ventilation. Their ICU stays lasted a median of 9 days (range 6–60). Notably, one patient (Patient 2) experienced a 60-day stay in the ICU, with 40 days spent on mechanical ventilation, attributed to the recurrent course of pulmonary haemorrhage. During this period, Patient 2, underwent 2 months of continuous ECMO therapy to support his respiratory function, and required 10 red blood cell transfusions. Patient 7 had frequent episodes of haemoptysis during the first month of hospitalization, with volumes ranging from 200 to 500 mL, and was managed by high-flow supplemental oxygen for 2 days. Patient 8 also required substantial oxygen support, with a maximum rate of 15 L/min for 9 days, before improving and requiring lower oxygen support. The other two patients required lower oxygen support.

**Figure 1:**
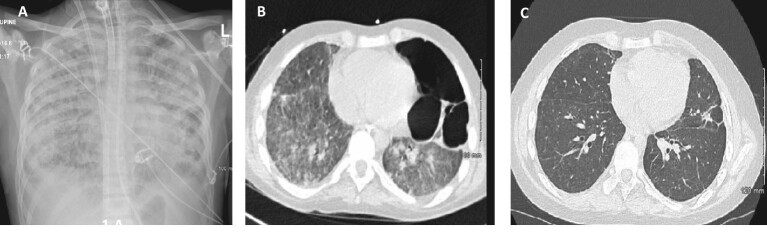
(**A**) Patient 2 after the first month: chest X-ray: bilateral interstitial infiltrates; (**B**) Patient 2 after 2 months: chest CT scan: diffuse ground-glass opacity of the lungs, residual left apical pneumothorax, loculated left lateral/basal hydropneumothorax; (**C**) Patient 2 after 6 months: chest CT scan: significantly improved diffuse ground glass opacification with subtle residual diffuse increased lung density, multiple diffuse bilateral predominantly sub-solid ground glass nodules, residual areas of scarring/atelectasis and traction bronchiectasis.

**Figure 2:**
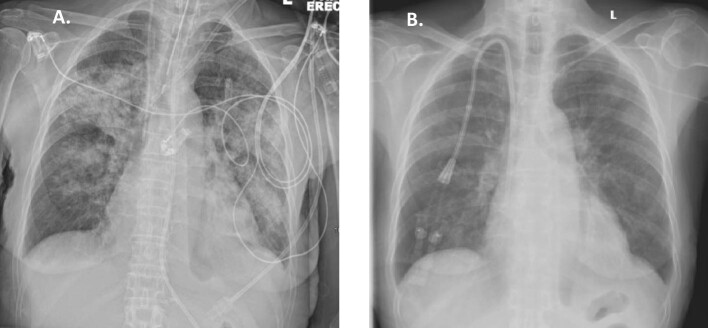
(**A**) Patient 3 at presentation: chest X-ray: bilateral consolidation; (**B**) Patient 3 after 10 days: chest X-ray: significant improvement.

**Figure 3:**
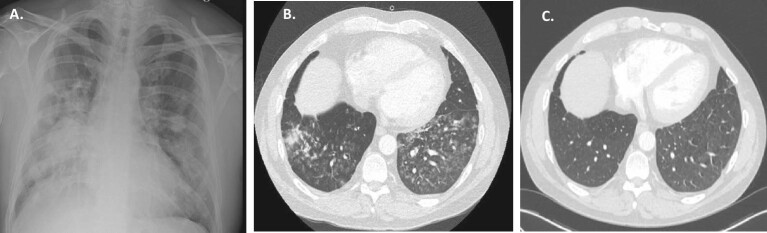
(**A**) Patient 7 at presentation: chest X-ray: bilateral consolidation; (**B**) Patient 7 at presentation: chest CT scan: diffuse ground-glass opacity of the lungs; (**C**) Patient 7 after 6 months: chest CT scan: significant improvement.

**Figure 4:**
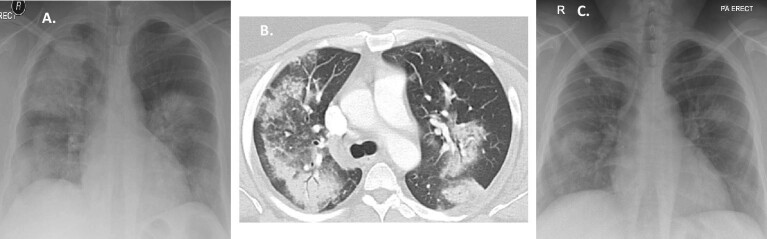
(**A**) Patient 8 at presentation: chest X-ray: bilateral consolidation; (**B**) Patient 8 at presentation: chest CT scan: diffuse ground-glass opacity of the lungs and areas of consolidation; (**C**) Patient 8 after 10 days: chest X-ray: significant improvement.

After the initiation of avacopan, all patients achieved independence from oxygen support after a median period of 6.5 days (range 0–40). Pulmonary haemorrhage resolved in all, and they experienced complete resolution of lung symptoms within a median of 10 days (range 2–40) (Figures 1–4). Of note, Patients 2 and 7, despite showing a resistant course of pulmonary haemorrhage for at least 1 month, having received rituximab, cyclophosphamide, high doses of steroids, PLEX and intravenous immunoglobulin (IVIG), achieved recovery within 30 days after the introduction of avacopan, and reduction in the cumulative steroid dose. All the patients were discharged. The initial hospitalization duration varied, reflecting differences in the severity and progression of pulmonary haemorrhage, with a median period of 13 days (range 5–84) from initiation of avacopan.

### Outcome

We recorded a median total follow-up of 6 months (range 2–13). All patients survived and achieved remission, and none experienced a relapse during the observational period. After 1 month of starting avacopan, the median prednisolone dose dropped to 5 mg/day (range 0–50), and impressively, three patients managed to completely discontinue their steroid use. There were no instances of rehospitalization during this period. During the total follow-up, among the patients with kidney involvement, two patients sustained normal kidney function, three showed improvement in eGFR, whereas two patients (Patients 3 and 6) remained dialysis dependent. Over the course of 1 year of treatment with avacopan, Patient 2 had complete radiological recovery (Fig. 1C) and functional improvement in forced vital capacity and total lung capacity. This patient's kidney function remained normal despite the prolonged ICU admission.

### Safety

The incidences of serious and non-serious adverse events are shown in Table 1. There were two serious infections, primarily of bacterial origin. In the case of Patient 2, a hospital-acquired pneumonia due to resistant bacteria and a possible fungal infection were observed during the first month of avacopan treatment while still on ICU. Patient 6, despite experiencing the resolution of lung symptoms, developed hospital-acquired pneumonia and an *Enterococcus* infection. These complications resulted in an extended hospitalization of 85 days. Three patients experienced non-serious infections, including COVID-19 infection without the need of re-hospitalization. Patient 4 underwent treatment with nirmatrelvir/ritonavir, and avacopan dose was reduced to 30 mg/day. Patient 5 developed a photosensitive rash after commencing avacopan, which was suspected to be a potential drug reaction, and discontinued avacopan after 3 days. Although the rash did not fully resolve with avacopan withdrawal, avacopan was not re-introduced as the patient was in sustained remission and remission maintenance treatment with rituximab monotherapy was considered sufficient. Patient 2 experienced neutropenia 3 months after starting avacopan which was temporarily discontinued and then re-introduced 1 month later.

## DISCUSSION

This retrospective case series presents data concerning the use of avacopan for hypoxic pulmonary haemorrhage due to AAV. As a life-threatening manifestation of AAV, the clinical utility of avacopan in this context is relevant because this drug has been approved for the treatment of AAV, yet patients with pulmonary haemorrhage requiring invasive pulmonary ventilation support were excluded from the clinical trials. We described eight cases with pulmonary haemorrhage characterized by hypoxia, four requiring ICU admission and mechanical ventilation. All patients recovered to leave hospital. These outcomes appear promising, particularly in terms of the rapid improvement in lung symptoms following the initiation of avacopan, even in previously treatment-resistant disease, compared with the high mortality of this presentation according to historic reports [10–12]. Additionally, avacopan allowed for a faster tapering of steroid doses, and may have improved the safety profile of the vasculitis treatment. This is relevant because early secondary infection due to prolonged and profound immunosuppression is a major cause of death in this population. In the PEXIVAS trial, the reported rate of serious infections in this subgroup was 41.4% [8].

The potential of C5a receptor antagonism has been assessed in two Phase 2 trials [13, 14] and one Phase 3 trial [9] in a total of 239 patients with AAV. These trials demonstrated that avacopan can serve as an alternative to glucocorticoids in combination with rituximab or cyclophosphamide, attractive for those patients at high risk of glucocorticoid toxicity. In addition, there was evidence from the ADVOCATE trial of superiority with avacopan with respect to sustained remission at 12 months, improvement in quality of life and fewer glucocorticoid related complications compared with a glucocorticoid regimen similar to the reduced-dose PEXIVAS regimen but with withdrawal at 21 weeks [9]. Interestingly, there was a notable and rapid improvement in the urine albumin–creatinine ratio by the end of the first month with avacopan treatment, showing an absolute difference of –40%, and better eGFR recovery at 12 months. The degree of proteinuria reflects the extent of glomerular lesions, and the quicker reduction in proteinuria suggests a swifter reduction in glomerular inflammation. It has also been shown that knock-out C5 mice, after injection of anti-MPO immunoglobulin G, did not develop albuminuria, haematuria or glomerular necrosis/crescents [15]. Additionally, earlier remission, based on Birmingham Vasculitis Activity Score, has been achievable with avacopan [9, 13, 14]. Considering the similarity in pathological lesions between capillaritis in ANCA-associated glomerulonephritis and pulmonary haemorrhage, it is plausible that this rapid reduction in disease activity with avacopan could also extend to the management of pulmonary haemorrhage [2]. In alignment with the hypothesis outlined above, our results have demonstrated that patients with hypoxic pulmonary haemorrhage who received avacopan, including patients requiring high levels of oxygen support, invasive mechanical ventilation or even extracorporeal support, and experiencing treatment-resistant pulmonary haemorrhage, were able to achieve independence from oxygen support within a total median time of 6.5 days (range 0–40). Moreover, within a median of 10 days (range 2–40) from avacopan initiation, complete resolution of pulmonary haemorrhage was achieved. We cannot attribute patient recovery to avacopan with confidence due to the use of other medications but note the apparent response to avacopan in refractory disease for two patients and the rapid effects seen in others. These improvements were not associated with any obvious increase in infection risk or other serious toxicity. It is also important to note that some of our patients received a higher cumulative dose of steroids compared with the patients in the ADVOCATE trial, and half received a combination of rituximab and cyclophosphamide [16]. Although these factors may be associated with a higher incidence of serious infections, the rate of serious infections was not higher than expected (25%). We also reported neutropenia occurring after 3 months of treatment, as has been previously documented, and physicians should be aware of this potential side-effect [9, 14]. It is also worth noting that only one patient permanently discontinued avacopan due to a rash, although this decision was primarily based on the patient's clinical improvement, rather than a definite attribution of the rash to avacopan. Given the limited experience of avacopan in this subgroup, it is valuable to thoroughly outline all potential side effects.

The PEXIVAS trial, which evaluated the use of PLEX and two regimens of oral glucocorticoids (standard and reduced) in patients with severe AAV, included 61 participants with severe pulmonary haemorrhage, presenting with hypoxia, among whom 29 required mechanical ventilation [7]. Although, in the total cohort the reduced dose steroid regimen was non-inferior in terms of the primary outcome death or end-stage kidney disease compared with the standard dose regimen, the subgroup of patients with severe pulmonary haemorrhage was characterized by higher and earlier mortality and there was a trend towards higher mortality in the reduced steroids subgroup [8]. Patients with severe/hypoxic pulmonary haemorrhage are more likely to receive treatments such as PLEX or high-dose steroids in an attempt to achieve remission. In this subgroup, characterized by a life-threatening condition, speed of treatment response is of essence. However, both PLEX and steroids carry an increased risk of infection [7, 9, 17]. Additionally, despite these interventions, in a historic report complete remission at 6 months was achieved in 68% of the patients requiring mechanical ventilation [10]. It is notable that despite six of our patients being in critical condition, all survived and achieved disease remission within a follow-up of 6 months (range 2–13). The median stay in the ICU lasted for 9 days (range 6–60), and in four cases, PLEX was performed. Additionally, in two patients during ICU, the nasogastric route was used, which has been tested for 5 weeks only in animal models [18]. The observed safety we herein report offers promising insights. Importantly, none of the patients required re-hospitalization after their initial discharge. After 1 month of avacopan treatment, the median steroid dose had reduced to 5 mg (range 0–50) and three patients discontinued their steroids. This observation underscores the potential for avacopan to facilitate rapid tapering of steroids, a subgroup in whom minimizing steroid-related side effects and complications is important.

Limitations of this study include its retrospective and observational nature, as well as a potential bias in avacopan selection. However, the case series includes all patients presenting with hypoxic pulmonary haemorrhage due to AAV in the participating centres who received avacopan. In addition, the attribution of avacopan to the outcomes is complicated by the time of avacopan initiation, variation in concomitant immunosuppressive treatment and PLEX. Acknowledging the heterogeneity in the evolution of pulmonary haemorrhage within our case series, it mirrors the diverse scenarios encountered in real-life management of hypoxic pulmonary haemorrhage. The descriptive presentation of all cases (Tables 1 and 2) offers practical reference points for clinical practice. Additionally, with the exception of the PEXIVAS trial, all previous reports of severe pulmonary haemorrhage in AAV are based on retrospective reviews, while the experience of avacopan in this subgroup has not yet been reported.

In conclusion, AAV patients with pulmonary haemorrhage typically require intensive immunosuppressive treatment regimens, including high dose of glucocorticoids and/or PLEX with cyclophosphamide and/or rituximab, in an attempt to rapidly reduce alveolar bleeding. This subgroup is characterized by a heightened risk of early mortality, due to infections and/or disease activity. Avacopan has shown potential to rapidly decrease renal disease activity, as well as achieve and sustain remission, thereby minimizing the necessity for steroids at sustained high doses. Despite the small number of cases that we report, the outcomes and the safety of avacopan in this subgroup, even in cases where extracorporeal support or mechanical ventilation was used, seems acceptable with all patients surviving to hospital discharge and last follow-up. We provide initial experience that can be considered when treating patients presenting with hypoxic pulmonary haemorrhage.

## Data Availability

The data that support the findings of this study are available from the corresponding author, upon reasonable request.
